# Real-world experience of safety and effectiveness of regorafenib for treatment of metastatic colorectal cancer, advanced gastrointestinal stromal tumors, and hepatocellular carcinoma: a post-marketing surveillance study in Korea

**DOI:** 10.7150/jca.74107

**Published:** 2022-09-21

**Authors:** Seung-Hoon Beom, Ki Beom Bae, Dae Young Zang, Joohee Bae, In Gyu Hwang, Hye Jin Kang, In Sook Woo, Byoung Yong Shim, Byung-Noe Bae, Jaekyung Cheon, Sang-Bo Oh, Joong-Bae Ahn

**Affiliations:** 1 Division of Medical Oncology, Department of Internal Medicine, Yonsei Cancer Center, Yonsei University College of Medicine, Seoul, Republic of Korea.; 2 Department of Surgery, Busan Paik Hospital, Inje University College of Medicine, Busan, Republic of Korea.; 3 Division of Hematology-Oncology, Department of Internal Medicine, Hallym University Sacred Heart Hospital, Hallym University College of Medicine, Anyang-si, Gyeonggi-do, Republic of Korea.; 4 Bayer Korea Ltd, Seoul, Republic of Korea.; 5 Division of Hemato-Oncology, Department of Internal Medicine, Chung-Ang University Hospital, Chung-Ang University College of Medicine, Seoul, Korea.; 6 Division of Hematology/Oncology, Department of Internal Medicine, Korea Cancer Center Hospital, Korea Institute of Radiological and Medical Sciences, Seoul, Korea.; 7 Division of Medical Oncology, Department of Internal Medicine, Yeouido St. Mary's Hospital, College of Medicine, The Catholic University of Korea, Seoul, Korea.; 8 Division of Medical Oncology, Department of Internal Medicine, St. Vincent's Hospital, College of Medicine, The Catholic University of Korea, Seoul, Korea; 9 Department of Surgery, Inje University Sanggye Paik Hospital, Seoul, Korea.; 10 Department of Medical Oncology, CHA Bundang Medical Center, CHA University School of Medicine, Seongnam, Korea.; 11 Division of Hematology-oncology, Department of Internal Medicine, Pusan National University Yangsan Hospital, Pusan National University School of Medicine, Yangsan, Korea.

**Keywords:** Regorafenib, Colorectal cancer, Gastrointestinal stromal tumors, Hepatocellular carcinoma, Real-world data, Post-marketing surveillance.

## Abstract

**Purpose:** This regulatory post-marketing surveillance (PMS) study was performed to evaluate the safety and effectiveness of regorafenib on Korean patients with colorectal cancer (CRC), gastrointestinal stromal tumors (GIST), and hepatocellular carcinoma (HCC) in a real-world clinical setting.

**Methods:** This PMS was conducted as a multi-center, prospective, observational study at 34 centers in Korea from August 2013 to August 2019. The primary objective was to evaluate the safety of regorafenib in real-world practice, with the secondary objective to investigate its effectiveness, including its overall response rate (ORR), progression-free survival (PFS), and overall survival (OS).

**Results:** In total, 301 patients were included in the analysis (254 patients with CRC, 14 patients with GIST, and 33 patients with HCC). The incidence rates of adverse events (AEs) were 85.0%, 78.6%, and 81.8% in patients with CRC, GIST, and HCC, respectively. The most frequent AE related to regorafenib in the three cancer types was palmar-plantar erythrodysesthesia syndrome (PPES). The ORRs of patients with CRC, GIST, and HCC were 4.7%, 0%, and 41.4%, respectively. The median PFS and OS were 2.1 and 6.1 months for CRC, respectively; 9.2 and 16.4 months for GIST, respectively; and 5.5 months and not estimated (NE) for HCC, respectively. Patients who experienced a dose modification or discontinuation of regorafenib showed significantly shorter median PFS and OS (2.2 vs. 2.6 months, respectively, *P* = 0.0335 for PFS; 5.3 vs. 8.5 months, respectively, *P* = 0.0010 for OS).

**Conclusion:** This PMS study, which is the largest surveillance study of CRC in Korea, found no newly identified safety concerns for patients who received regorafenib in the real-world setting. Additionally, the results of this study were consisted with those previously reported in phase III trials.

## Introduction

Regorafenib (Bayer HealthCare Pharmaceuticals Inc., Berlin, Germany) is an oral multi-kinase inhibitor; the recommended daily dose is 160 mg (four 40 mg tablets, taken orally) 1. In 2012, the United States of America Food and Drug Administration (FDA) approved regorafenib for previously treated patients with metastatic colorectal cancer (CRC). In 2013, the FDA also approved regorafenib as a third-line treatment for patients with locally advanced, unresectable gastrointestinal stromal tumors (GIST) who were previously treated with imatinib mesylate and sunitinib malate; in 2017, they approved it as a second-line treatment for patients with hepatocellular carcinoma (HCC) who were previously treated with sorafenib 1.

CRC is the third most common cancer globally and has the second-highest mortality rate in the world 2. In particular, Korea had the second-highest incidence of CRC in the world in 2018 3, 4. The standard treatment for patients with CRC includes fluoropyrimidine, oxaliplatin, irinotecan, and anti-vascular endothelial growth factor (VEGF); an anti-epidermal growth factor receptor (EGFR) therapy is also used for Kirsten rat sarcoma viral oncogene homolog (KRAS) wild type CRC 5. Studies have been conducted to establish regorafenib as a treatment option for patients with metastatic CRC who have been previously treated with standard therapies 5,6. The CORRECT and CONCUR trials, which were phase III clinical trials of the efficacy of regorafenib versus a matching placebo as a treatment for CRC, showed improved overall survival (OS; hazard ratio = 0.77, 95% CI 0.64-0.94, one-sided *P* = 0.0052 in CORRECT; hazard ratio = 0.55, 95% CI 0.40-0.77, one-sided *P* = 0.0016 in CONCUR) 5,6

GIST is a common sarcoma that forms in the gastrointestinal tract. If complete surgical resection of GIST is difficult, prognosis is unfavorable; targeted therapies are recommended for treatment for such cases 7. Most patients with metastatic GIST are treated with imatinib and sunitinib, but an additional treatment is required if these drugs fail 8. In the phase III GRID trial, which was conducted on patients with unresectable or metastatic GIST after standard therapies, regorafenib improved the primary endpoint of PFS, compared to the placebo group (hazard ratio = 0.27, 95% CI 0.19-0.39, *P* <0.0001) 8.

HCC is a common primary liver cancer and is the sixth most common cancer worldwide 2. Liver resection and liver transplantation are the primary treatments for liver cancer 9, with systemic treatment being carried out as a non-surgical alternative for patients who cannot be treated using locoregional therapy 10. Sorafenib is widely used as a first-line systemic treatment for HCC; however, a second-line systemic treatment was needed 11. The phase III RESORCE trial showed that regorafenib achieved an improved OS in patients with HCC who had been previously treated with sorafenib (hazard ratio = 0.63, 95% CI 0.50-0.79, one-sided *P* <0.0001) 10.

Although these phase III trials assessed both the safety and efficacy of regorafenib 5, 6, 8, 10, the safety profile of regorafenib in routine clinical practice is not evaluated. It is necessary to collect real-world evidence under various clinical conditions to identify regorafenib's safety profile, and to determine how the prognosis changes in response to dose modification or discontinuation in actual clinical practice. Thus, this study aimed to evaluate the safety and effectiveness of regorafenib in a real-world routine setting by analyzing its usage in the treatment of Korean patients with CRC, GIST, and HCC.

## Materials and Methods

### Study design and patients

This regulatory post-marketing surveillance (PMS) was a multi-center, prospective observational study. Following the approval of regorafenib in August 2013 by the Korean Ministry of Food and Drug Safety (MFDS), this PMS study was conducted at 34 Korean hospitals between August 2013 to August 2019 to collect information on the safety and effectiveness of regorafenib under routine clinical practice in Korea (Trial Registration ID: NCT02106858).

Patients were included in the study if they were prescribed regorafenib for the first time in routine clinical practice under the following approved label by MFDS: 1) those with metastatic CRC who have previously been treated with fluoropyrimidine-based chemotherapy, anti-VEGF treatment, and anti-EGFR treatment (in the case of RAS wild type); 2) those with metastatic or unresectable locally advanced GIST previously treated with imatinib mesylate and sunitinib malate; and 3) those with HCC previously treated with sorafenib. However, patients who were participating in any investigational programs with interventions outside of routine clinical practice were excluded. Regorafenib treatment was continued until disease progression or unacceptable toxicity, and the last visit of the patient was performed at 30 days after treatment termination.

This study was performed in accordance with the World Medical Association Declaration of Helsinki. This study was also reviewed by the MFDS as a regulatory requirement in Korea and approved by the institutional review boards (IRBs) of the participating centers. Informed consent was obtained from all patients, in accordance with the study protocol.

### Study outcomes and measurements

Demographic data, including age, sex, clinical characteristics (e.g., diagnostic information about metastasis sites, prior therapies, and Eastern cooperative oncology group performance status [ECOG PS]), were obtained from each patient. All adverse events (AEs) were collected from the date that the patient signed the informed consent to 30 days after the termination of treatment; they were described using preferred terms (PTs) according to the Medical Dictionary for Regulatory Activities (MedDRA) version 21.0. The severities of AE were assessed according to National Cancer Institute Common Terminology Criteria for Adverse Events (NCI CTCAE) 4.03, and their relationships with regorafenib were assessed by the investigators. Adverse drug reactions (ADRs) were defined as any AE for which causal relationship with regorafenib cannot be excluded. Also, the incidences of AEs were additionally investigated for the special interest populations from a safety standpoint (patients who were elderly; who had hepatic, renal, or cardiovascular disorders; or who had other concomitant diseases).

Effectiveness analyses included the objective response rate (ORR), PFS, and OS. Tumor response assessments were conducted during the physician's routine practice based on Response Evaluation Criteria in Solid Tumor (RECIST) criteria version 1.1 using radiologic evaluation, including computed tomography (CT). Investigators' clinical assessments were used in cases wherein radiological examinations cannot be performed. Tumor response assessment was classified into “Complete response (CR),” “Partial response (PR),” “Stable disease (SD),” and “Progressive disease (PD).” ORR was defined as “CR” and “PR,” and disease control rate (DCR) was defined as “CR,” “PR,” and “SD.” ORR and DCR were recorded from the time of the first regorafenib administration to the last follow-up. PFS was defined as the time from the first regorafenib administration to the first documented “PD” or death from any cause, whichever occurred first, and OS was defined as the time from the first study drug administration to death from any cause.

### Statistical analysis and methods

Continuous variables are presented as descriptive statistics, including means and standard deviations, whereas categorical variables are expressed as frequencies and ratios. The safety profile of regorafenib was summarized by the numbers and percentages of patients with AEs and ADRs for all grades, and separately for those of grades ≥3. ORR was calculated as the numbers and percentages of patients among patients whose response were evaluated. PFS and OS were estimated by tumor type using the Kaplan-Meier method. Log rank tests were used to compare the survival rates between the subgroups. As an exploratory analysis, PFS, OS, ORR, and DCR between patients who had dose modification or permanent discontinuation due to AEs and who did not were compared using either Fisher's exact tests or Chi-square tests. Univariate logistic regression was performed to identify clinical parameters that affect CRC patients with or without dose modification or discontinuation. Statistical analyses were performed using SAS version 9.4 (SAS Institute, Cary NC, USA).

## Results

### Baseline characteristics

**Fig 1:**
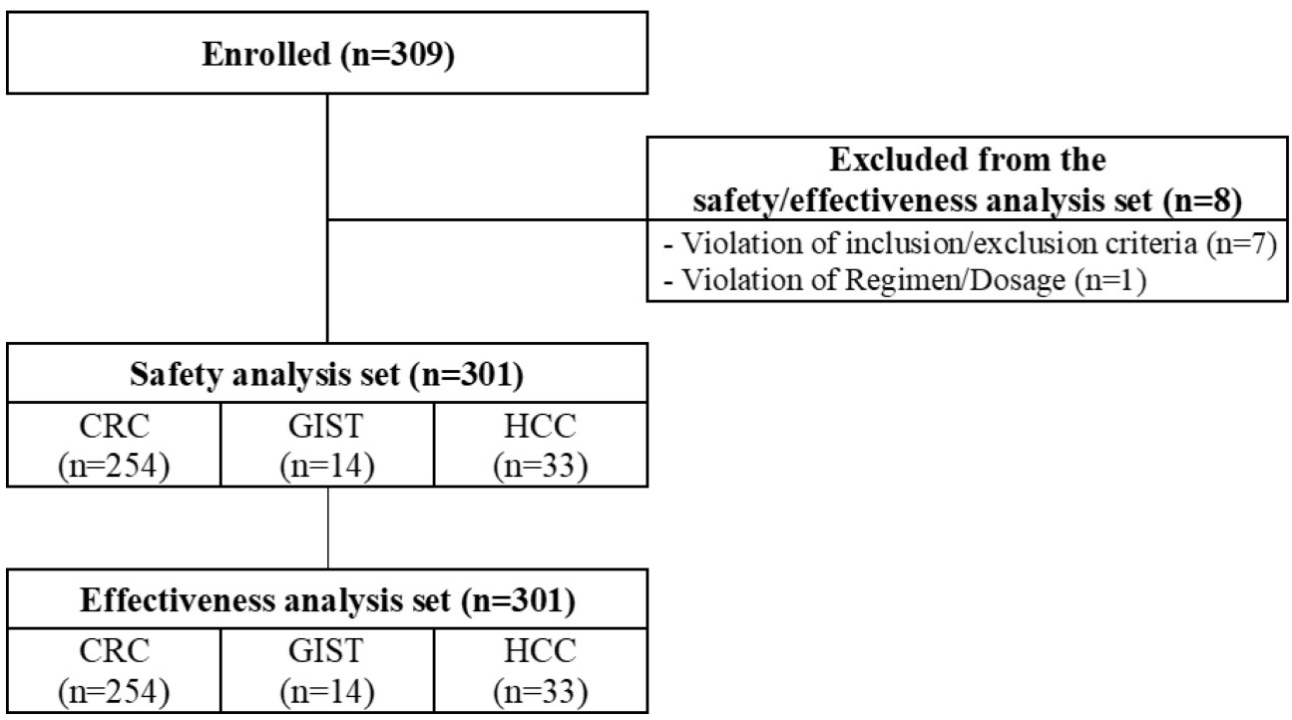
** Patient disposition.** CRC, colorectal cancer; GIST, gastrointestinal stromal tumors; HCC, hepatocellular carcinoma.

In total, 309 patients were enrolled, with safety and effectiveness evaluations being conducted on 301 patients (254 patients with CRC, 14 patients with GIST, and 33 patients with HCC). The baseline characteristics and disposition of these patients are presented in Table 1 and Fig. 1, respectively. Males made up 52.0, 78.6, and 84.9% of patients with CRC, GIST, and HCC, respectively. The median ages of patients with CRC, GIST, and HCC were 58 years (range = 31-82 years), 56 years (range = 40-68 years), and 63 years (range = 53-76 years), respectively. The most common metastasis site for CRC and GIST patients was the liver (66.5 and 71.4%, respectively), whereas that for HCC patients was the lungs (30.3%). More than half of the CRC and GIST patients received surgery as a prior therapy (78.7 and 78.6%, respectively), while 18.2% of HCC patients received surgery or radiotherapy. Furthermore, 89.0% of CRC patients received four or more prior treatment lines, while 64.3 and 81.8% of GIST and HCC patients, respectively, received one to two prior treatment lines. Patients with CRC previously received cytotoxic chemotherapy (99.2%), anti-VEGF biologics (93.7%), or anti-EGFR biologics (34.7%) as prior treatments, while majority of patients with HCC and all patients with GIST received tyrosine kinase inhibitors (Table S1). Patients with CRC, GIST, and HCC had an ECOG PS of 0 (15.0, 14.3, and 24.2%, respectively) or 1 (40.2, 35.7, and 18.2%, respectively). All patients with HCC had a Child-Pugh class of A.

### Treatment duration and dose

The treatment duration and dosing of regorafenib are shown in Table 2. The median treatment durations were 1.6, 4.2, and 2.5 months for patients with CRC, GIST, and HCC, respectively. More than half of the patients (64.4% of patients with CRC, 53.9% of patients with GIST, and 87.9% of patients with HCC) of all cancer types received 160 mg daily. The mean daily doses were 147.1, 144.8, and 153.8 mg for patients with CRC, GIST, and HCC, respectively.

### Safety

The incidence rates of AEs are summarized in Table 3, with AEs that occurred in >1% of the patients are listed in Tables S2-S4. During the study period, 216 (85.0%) patients with CRC, 11 (78.6%) patients with GIST, and 27 (81.8%) patients with HCC reported at least one AE. The most frequent AE in all three indications was palmar-plantar erythrodysesthesia syndrome (PPES) that were identified to be drug related (28.0% of patients with CRC, 35.7% of patients with GIST, and 15.2% of patients with HCC). The most common ADRs of grade ≥3 in patients with CRC were PPES (n = 13, 5.1%), followed by anemia (n = 5, 2.0%) and asthenia (n = 4, 1.6%). PPES (14.3%) was recorded in patients with GIST, while no ADR of grade ≥3 was recorded in patients with HCC.

Unexpected ADRs were reported in 15.8% (n = 40) of patients with CRC, 28.6% (n = 4) of patients with GIST, and 15.2% (n = 5) of patients with HCC. The most common unexpected ADRs were dyspepsia (n = 5), followed by blister (n = 3), dyspnoea (n = 2), productive cough (n = 2), and hyperkeratosis (n = 2; Tables S2-S4).

Seventy-one patients (23.6%) had a dose reduction, while 66 (21.9%) had a dose interruption due to AEs. Treatment was permanently discontinued in 74 patients (24.6%) because of AEs (Table S5). The most frequent AEs that resulted in dose modification were PPES (13.0%), asthenia (3.7%), and rash (2.3%). The most frequent AEs leading to permanent discontinuation were asthenia (4.0%), PPES (3.0%), and abdominal pain (2.3%).

In addition, no statistically significant differences in AE incidences were observed between the patient in the special interest group (patients who were elderly; who had hepatic, renal, or cardiovascular disorders; or who had other concomitant diseases) and the general patient group (Table S6).

### Effectiveness

The overall responses are summarized in Table 5. The ORR and DCR were 4.7 and 29.5%, respectively, for patients with CRC; 0.0 and 77.8%, respectively, for patients with GIST; and 41.4 and 79.3%, respectively, for patients with HCC.

**Fig 2:**
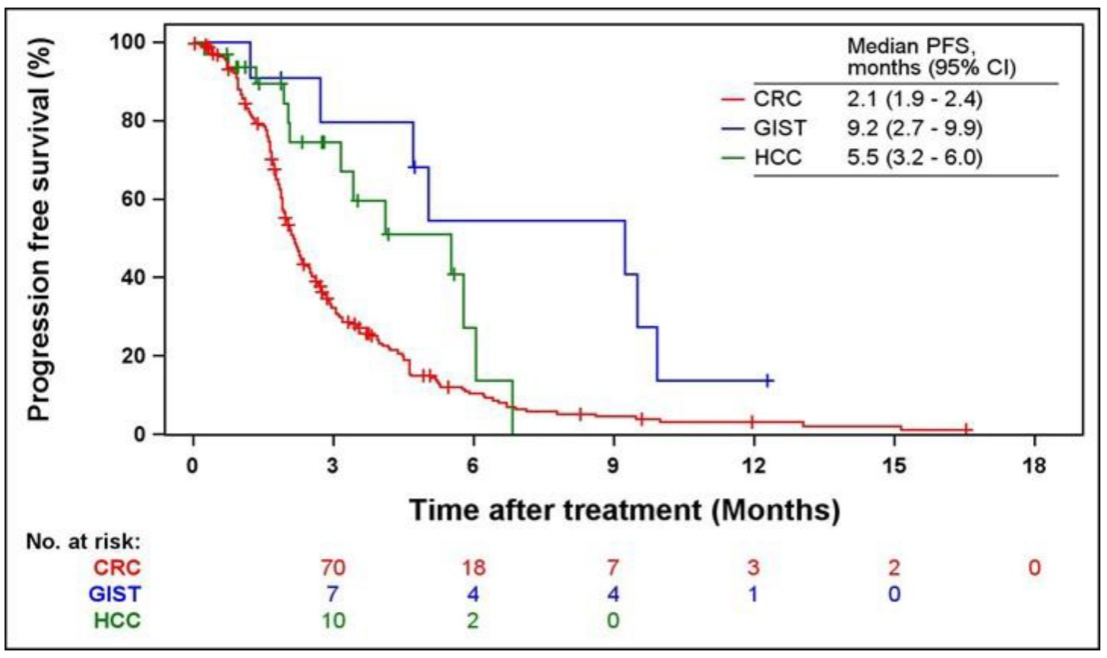
** Kaplan-Meier curves of PFS from the first regorafenib administration.** PFS, progression-free survival; CRC, colorectal cancer; GIST, gastrointestinal stromal tumors; HCC, hepatocellular carcinoma.

**Fig 3:**
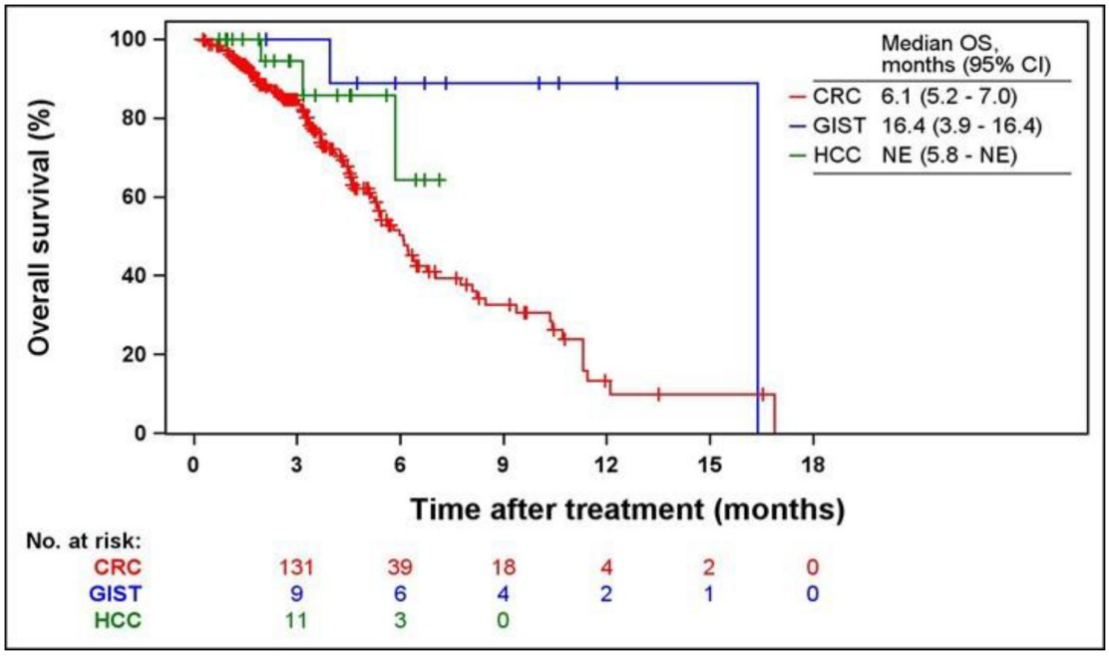
** Kaplan-Meier curves of OS from the first regorafenib administration.** OS, overall survival; CRC, colorectal cancer; GIST, gastrointestinal stromal tumors; HCC, hepatocellular carcinoma; NE, not estimated.

Kaplan-Meier curves of PFS and OS according to indications are shown in Figs. 2 and 3. The median PFS were 2.1 (95% CI = 1.9-2.4), 9.2 (95% CI = 2.7-9.9), and 5.5 months (95% CI = 3.2-6.0) in patients with CRC, GIST, and HCC, respectively. The median OS was 6.1 months (95% CI = 5.2-7.0) and 16.4 months (95% CI = 3.9-16.4) in patients with CRC and GIST, respectively. The median OS was not estimated (NE) in patients with HCC (95% CI = 5.8-NE).

The median PFS and OS were significantly shorter in patients who experienced a dose modification or discontinuation of regorafenib due to AEs (2.2 vs. 2.6 months, *P* = 0.0335 for PFS; 5.3 vs. 8.5 months, *P* = 0.0010 for OS) (Table 6). More than half of the patients with dose modification or discontinuation (74/143 patients) permanently discontinued their treatments. In addition, no significant differences were observed between clinical parameters of CRC patients with or without dose modification or discontinuation (Table S7).

## Discussion

This PMS study was conducted on patients who were prescribed regorafenib in a real-world clinical setting to evaluate the safety of regorafenib. Most of the patients enrolled in this study had CRC, with smaller numbers of patients with HCC and GIST. To our knowledge, this study is the largest real-world study on Korean patients with CRC who were treated with regorafenib.

While this study was conducted under routine clinical practice, the safety profile and effectiveness of regorafenib for patients with CRC were found to be similar to the results of the CORRECT phase III trial 5. The incidence of AEs recorded in the present study was lower than that in CORRECT. In the present study, 85% of patients with CRC experienced at least one AE, while 100% of the patients in the CORRECT phase III trial had an AE 5. PPES was the most frequent AE of grade ≥3 in both studies; however, the incidence rate of PPES was lower in the present study (5% vs. 17%) 5. Likewise, PPES was the most common ADR in a Japanese PMS study; however, the incidence rate of PPES was relatively lower in the present study (28% vs. 58%) 12. Furthermore, the median PFS and OS of patients with CRC in this study were 2.1 and 6.1 months, respectively; similarly, those in the CORRECT were 1.9 (interquartile range [IQR] = 1.6-3.9) and 6.4 months (IQR = 1.6-3.9), respectively 5. It is meaningful that the results of the present study were consistent with those of the phase III trial. Unlike the phase III trial, which included only patients with ECOG PS 0-1, the present study showed that 6.3% of the patients with CRC had an ECOG PS of 2. Furthermore, most patients in the present study were more heavily treated before regorafenib. In the present study, most patients (89.0%) received four or more prior treatment lines, compared to that in the CORRECT (49%) 5. The number of patients that received regorafenib as a later treatment line was also higher in the present study compared to the large-scale prospective observational CORRELATE study 13.

In the phase III trials, dose reduction or interruption due to AE occurred for approximately 70% of patients with CRC, GIST, and HCC, with treatment discontinuation occurring for 6-25% of patients 5, 8, 10, In the present study, AEs that resulted in dose reduction (24%) or interruption (22%) were less frequent; while those that resulted in discontinuation (25%) were similar. This suggests that AEs were well-managed in the real-world clinical setting. On the other hand, patients with reduced dosage of or discontinued regorafenib had a significantly lower survival benefit than those who continued with treatment. Therefore, continuous treatment of regorafenib while managing AEs is necessary. Aside from these observations, no new safety signals were observed.

Variations on the treatment duration among different cancer types were observed. Median treatment duration was relatively shorter in CRC and longer in GIST, which were similar to those in the phase III trials. The median duration of regorafenib treatment were 1.7, 5.3, and 3.6 months in CRC, GIST, and HCC in phase III trials, unlike those of placebo treatment were all similarly less than 2 months 5, 8, 10. These observations may be due to different cancer characteristics and difference in the effectiveness of regorafenib in different cancer types. Likewise, the PFS and OS also appeared differently for each cancer type; however, the effectiveness of regorafenib in different cancer types was not compared due to the small number of patients with GIST and HCC.

One limitation of the present study is the insufficient numbers of patients with HCC or GIST, which made comparison with previously reported phase III trials difficult. Although the PMS study should include all registry-approved indications, smaller numbers of patients with HCC and GIST were enrolled because GIST is a rare tumor, while regorafenib for HCC was the last to be approved by the Korean MFDS in 2017. However, it is not necessary to conduct further studies with more HCC patients regarding the effectiveness of regorafenib, because the effectiveness of regorafenib in HCC has been confirmed through the global large-scale prospective, observational REFINE study 14, 15 and a larger real-world study on 440 HCC patients in Korea 16. Another limitation is that a longer survival follow-up was not conducted after end of the study. As patients with HCC were enrolled by the end of study, median follow-up duration was 2.3 months, which is not sufficient to estimate the median OS of HCC.

Consequently, this PMS study, which is the first to evaluate safety of regorafenib on a prospective and large scale in real world in Korean patients with CRC, GIST and HCC, found no new concerns in the safety profile of regorafenib, compared to the current marketing authorization in Korea. The effectiveness of regorafenib in Korean patients with CRC was similar in a real-world setting to that in the previously reported phase III trials.

## Supplementary Material

Supplementary tables.

## Figures and Tables

**Table 1:** Baseline characteristics.

Variable	CRC (N=254) No. (%)	GIST (N=14) No. (%)	HCC (N=33) No. (%)
Sex			
Male	132 (52.0)	11 (78.6)	28 (84.9)
Female	122 (48.0)	3 (21.4)	5 (15.2)
Age (y), median (range)	58.0 (31.0-82.0)	55.5 (40.0-68.0)	63.0 (53.0-76.0)
Metastasis site^(a)^			
Liver	169 (66.5)	10 (71.4)	2 (6.1)
Lung	133 (52.4)	1 (7.1)	10 (30.3)
Bone	26 (10.2)	0 (0.0)	4 (12.1)
Spinal cord	6 (2.4)	0 (0.0)	0 (0.0)
Skin/Soft tissue	1 (0.4)	0 (0.0)	0 (0.0)
Brain	7 (2.8)	0 (0.0)	1 (3.0)
Gastrointestinal	4 (1.6)	0 (0.0)	0 (0.0)
Distant LNs	78 (30.7)	0 (0.0)	1 (3.0)
Local regional LNs	23 (9.1)	0 (0.0)	2 (6.1)
Other	67 (26.4)	5 (35.7)	2 (6.1)
Prior therapy^(b)^			
Radiotherapy	72 (28.4)	1 (7.1)	6 (18.2)
Surgery	200 (78.7)	11 (78.6)	6 (18.2)
Prior treatment lines^ (c)^			
1-2	8 (3.2)	9 (64.3)	27 (81.8)
3	20 (7.9)	1 (7.1)	1 (3.0)
≥ 4	226 (89.0)	4 (28.6)	5 (15.2)
ECOG PS^(b)^			
0	38 (15.0)	2 (14.3)	8 (24.2)
1	102 (40.2)	5 (35.7)	6 (18.2)
2	16 (6.3)	1 (7.1)	0 (0.0)
3	0 (0.0)	0 (0.0)	1 (3.0)
Missing value	98 (38.6)	6 (42.9)	18 (54.5)

CRC, colorectal cancer; GIST, gastrointestinal stromal tumors; HCC, hepatocellular carcinoma; LN, lymph node; ECOG PS, Eastern cooperative oncology group performance status. **(A)**Values were overlap collected. **(B)**Missing values were excluded. **(C)**In HCC, transarterial chemoembolization may be included.

**Table 2:** Treatment profile of regorafenib.

Variable	CRC (N=254) No. (%)	GIST (N=14) No. (%)	HCC (N=33) No. (%)
Treatment duration (months)^(a)^			
Mean ± STD	2.2 ± 2.1	4.6 ± 3.0	2.8 ± 1.8
Median	1.6	4.2	2.5
Min, Max	0.03, 14.46	0.89, 9.87	0.03, 6.00
Missing value	1	1	0
Daily dose of regorafenib^(a)^			
< 80 mg/days	0 (0.0)	0 (0.00)	0 (0.00)
≥ 80 mg/day - < 120 mg/day	20 (7.9)	2 (15.4)	2 (6.1)
≥ 120 mg/day - < 160 mg/day	70 (27.7)	4 (30.8)	2 (6.1)
160 mg/day	163 (64.4)	7 (53.9)	29 (87.9)
Missing value	1	1	0

CRC, colorectal cancer; GIST, gastrointestinal stromal tumors; HCC, hepatocellular carcinoma; STD, standard deviation. **(A)**Missing values were excluded.

**Table 3:** Overall summary of AEs with regorafenib use.

Variable	CRC (N=254)	GIST (N=14)	HCC (N=33)
	No. (%)	No. (%)	No. (%)
AEs	216 (85.0)	11 (78.6)	27 (81.8)
ADRs	163 (64.2)	10 (71.4)	17 (51.5)
Serious AEs	87 (34.3)	3 (21.4)	9 (27.3)
Serious ADRs	31 (12.2)	1 (7.1)	1 (3.0)
Unexpected AEs	110 (43.3)	6 (42.9)	14 (42.4)
Unexpected ADRs	40 (15.8)	4 (28.6)	5 (15.2)

CRC, colorectal cancer; GIST, gastrointestinal stromal tumors; HCC, hepatocellular carcinoma; AE, adverse event, ADR, adverse drug reaction.

**Table 4:** Incidence rates of the most common AEs and ADRs for patients with CRC (N=254).

	AE		ADR	
	Any grade	≥ Grade 3	Any grade	≥ Grade 3
	No. (%)	No. (%)	No. (%)	No. (%)
Palmar-plantar erythrodysesthesia syndrome	71 (28.0)	13 (5.1)	71 (28.0)	13 (5.1)
Asthenia	29 (11.4)	10 (3.9)	17 (6.7)	4 (1.6)
Abdominal pain	24 (9.5)	4 (1.6)	4 (1.6)	0 (0.0)
Aspartate aminotransferase increased	24 (9.5)	2 (0.8)	15 (5.9)	1 (0.4)
Decreased appetite	24 (9.5)	3 (1.2)	15 (5.9)	1 (0.4)
Diarrhea	21 (8.3)	2 (0.8)	20 (7.9)	2 (0.8)
Pyrexia	21 (8.3)	0 (0.0)	2 (0.8)	0 (0.0)
Rash	20 (7.9)	3 (1.2)	16 (6.3)	2 (0.8)
Alanine aminotransferase increased	15 (5.9)	2 (0.8)	7 (2.8)	1 (0.4)
Stomatitis	15 (5.9)	2 (0.8)	13 (5.1)	2 (0.8)

AE, adverse event; ADR, adverse drug reaction.

**Table 5:** Overall responses of patients.

Variable	CRC (N=254)	GIST (N=14)	HCC (N=33)
Evaluated, n (%)*	N=193	N=9	N=29
CR	0 (0.0)	0 (0.0)	0 (0.0)
PR	9 (4.7)	0 (0.0)	12 (41.4)
SD	48 (24.9)	7 (77.8)	11 (37.9)
PD	136 (70.5)	2 (22.2)	6 (20.7)
ORR^a)^	9 (4.7)	0 (0.0)	12 (41.4)
DCR^b)^	57 (29.5)	7 (77.8)	23 (79.3)
Not evaluated (n)	61	5	4

CRC, colorectal cancer; GIST, gastrointestinal stromal tumors; HCC, hepatocellular carcinoma; CR, complete response; PR, partial response; SD, stable disease; PD, progressive disease; ORR, overall response rate; DCR, disease control rate. * Patients whose response were not evaluated were excluded ^a)^ ORR was defined as the number of subjects with best overall response of CR and PR. ^b)^ DCR was defined as the number of subjects with best overall response of CR, PR, and SD.

**Table 6:** PFS, OS, ORR, and DCR according to dose modification or discontinuation of regorafenib administration.

	CRC			Total		
	Dose modification or discontinuation*			Dose modification or discontinuation*		
	Yes (N=124)	No (N=130)	P-value	Yes (N=143)	No (N=158)	P-value
PFS, median (months)	2.1	2.2	0.2718^a)^	2.2	2.6	0.0335^a)^
OS, median (months)	5.2	6.4	0.0173^a)^	5.3	8.5	0.0010^a)^
ORR, n(%)^d,e)^	2 (2.1)	7 (7.1)	0.1707^b)^	4 (3.7)	17 (13.7)	0.0086^c)^
DCR, n(%)^f)^	25 (26.6)	32 (32.3)	0.3833^c)^	33 (30.8)	54 (43.5)	0.0469^c)^

CRC, colorectal cancer; GIST, gastrointestinal stromal tumors; HCC, hepatocellular carcinoma; PFS, progression-free survival; OS, overall survival; ORR, overall response rate; DCR, disease control rate. *Dose modification or discontinuation included dose change, dose interruption, and permanent discontinuation ^a)^ P-value by Log rank test. ^b)^ P-value by Fisher's exact test. ^c)^ P-value by Chi-square test. ^d)^ Patients whose response was not evaluated were excluded ^e)^ ORR was defined as the number of subjects with best overall response of CR and PR. ^f)^ DCR was defined as the number of subjects with best overall response of CR, PR, and SD.

## Abbreviations

ADRs: adverse drug reactions
AEs: adverse events
CR: complete response
CRC: colorectal cancer
CT: computed tomography
DCR: disease control rate
ECOG PS: eastern cooperative oncology group performance status
EGFR: epidermal growth factor receptor
FDA: Food and Drug Administration
GIST: gastrointestinal stromal tumors
HCC: hepatocellular carcinoma
IRB: institutional review board
KRAS: kirsten rat sarcoma viral oncogene homolog
MedDRA: Medical Dictionary for Regulatory Activities
MFDS: Ministry of Food and Drug Safety
NCI CTCAE: National Cancer Institute Common Terminology Criteria for Adverse Events
NE: not estimated
ORR: overall response rate
OS: overall survival
PD: progressive disease
PFS: progression-free survival
PMS: post-marketing surveillance
PPES: palmar-plantar erythrodysesthesia syndrome
PR: partial response
PT: preferred term
RECIST: Response Evaluation Criteria in Solid Tumor
SD: stable disease
VEGF: vascular endothelial growth factor
