# Supplementary material for: Real-world experience of safety and effectiveness of regorafenib for treatment of metastatic colorectal cancer, advanced gastrointestinal stromal tumors, and hepatocellular carcinoma: a post-marketing surveillance study in Korea
Source: J Cancer. 2022 Sep 21;13(13):3396–403. doi: 10.7150/jca.74107 (PMC9608207; doi:10.7150/jca.74107)
Supplement: Supplementary file 1 — Supplementary tables. [file jcav13p3396s1.pdf]

Table S1. Prior anticancer agents

	CRC (N=254)	GIST (N=14)	HCC (N=33)
	No. of patient<br>N (%)	No. of patient<br>N (%)	No. of patient<br>N (%)
Cytotoxic chemotherapy	252 (99.2)	0 (0.0)	9 (27.3)
anti-VEGF biologics	238 (93.7)	0 (0.0)	0 (0.0)
anti-EGFR biologics	88 (34.7)	0 (0.0)	0 (0.0)
TKIs	1 (0.4)	14 (100.0)	32 (97.0)
Investigational drug*	4 (1.6)	0 (0.0)	0 (0.0)

* Patients who participated in other clinical trials

Table S2. Incidence rates of AEs and ADRs for CRC patients (>1%)

	CRC (N=254)			
	AE		ADR	
	Any grade No. (%)	≥ Grade 3 No. (%)	Any grade No. (%)	≥ Grade 3 No. (%)
**Clinical adverse event**				
Palmar-plantar erythrodysaesthesia syndrome	71 (28.0)	13 (5.1)	71 (28.0)	13 (5.1)
Asthenia	29 (11.4)	10 (3.9)	17 (6.7)	4 (1.6)
Abdominal pain	24 (9.5)	4 (1.6)	4 (1.6)	0 (0.0)
Decreased appetite	24 (9.5)	3 (1.2)	15 (5.9)	1 (0.4)
Diarrhoea	21 (8.3)	2 (0.8)	20 (7.9)	2 (0.8)
Pyrexia	21 (8.3)	0 (0.0)	2 (0.8)	0 (0.0)
Rash	20 (7.9)	3 (1.2)	16 (6.3)	2 (0.8)
Stomatitis	15 (5.9)	2 (0.8)	13 (5.1)	2 (0.8)
Back pain	12 (4.7)	4 (1.6)	1 (0.4)	0 (0.0)
Constipation*	12 (4.7)	0 (0.0)	1 (0.4)	0 (0.0)
Vomiting	12 (4.7)	3 (1.2)	6 (2.4)	0 (0.0)
Nausea	10 (3.9)	1 (0.4)	5 (2.0)	0 (0.0)
Dyspepsia*	9 (3.5)	0 (0.0)	5 (2.0)	0 (0.0)
Cough*	8 (3.2)	2 (0.8)	1 (0.4)	0 (0.0)
Dyspnoea*	8 (3.2)	1 (0.4)	2 (0.8)	1 (0.4)
Fatigue	8 (3.2)	3 (1.2)	5 (2.0)	2 (0.8)

Oedema peripheral*	7 (2.8)	0 (0.0)	1 (0.4)	0 (0.0)
Abdominal pain upper	6 (2.4)	0 (0.0)	0 (0.0)	0 (0.0)
Jaundice*	6 (2.4)	2 (0.8)	1 (0.4)	1 (0.4)
Musculoskeletal pain	6 (2.4)	2 (0.8)	0 (0.0)	0 (0.0)
Pain in extremity	6 (2.4)	0 (0.0)	2 (0.8)	0 (0.0)
Abdominal distension*	5 (2.0)	3 (1.2)	1 (0.4)	0 (0.0)
Myalgia	5 (2.0)	0 (0.0)	4 (1.6)	0 (0.0)
Pneumonia	5 (2.0)	1 (0.4)	0 (0.0)	0 (0.0)
Productive cough*	5 (2.0)	0 (0.0)	2 (0.8)	0 (0.0)
Sepsis	5 (2.0)	4 (1.6)	1 (0.4)	1 (0.4)
Urinary tract infection	5 (2.0)	3 (1.2)	0 (0.0)	0 (0.0)
Dysphonia	4 (1.6)	0 (0.0)	2 (0.8)	0 (0.0)
Flank pain	4 (1.6)	0 (0.0)	0 (0.0)	0 (0.0)
Hypertension	4 (1.6)	1 (0.4)	1 (0.4)	1 (0.39)
Hypophagia	4 (1.6)	0 (0.0)	1 (0.4)	0 (0.0)
Oropharyngeal pain	4 (1.6)	0 (0.0)	1 (0.4)	0 (0.0)
Pain	4 (1.6)	0 (0.0)	1 (0.4)	0 (0.0)
Peripheral sensory neuropathy	4 (1.6)	0 (0.0)	4 (1.6)	0 (0.0)
Blister*	3 (1.2)	0 (0.0)	3 (1.2)	0 (0.0)
Cancer pain	3 (1.2)	0 (0.0)	2 (0.8)	0 (0.0)
Haematuria	3 (1.2)	1 (0.4)	0 (0.0)	0 (0.0)
Headache	3 (1.2)	0 (0.0)	1 (0.4)	0 (0.0)
Hepatitis	3 (1.2)	1 (0.4)	2 (0.8)	0 (0.0)

Hiccups*	3 (1.2)	0 (0.0)	0 (0.0)	0 (0.0)
Insomnia*	3 (1.2)	0 (0.0)	1 (0.4)	0 (0.0)
Mucosal inflammation	3 (1.2)	0 (0.0)	2 (0.8)	0 (0.0)
Muscular weakness*	3 (1.2)	0 (0.0)	1 (0.4)	0 (0.0)
Pleural effusion*	3 (1.2)	2 (0.8)	0 (0.0)	0 (0.0)
Procedural pain	3 (1.2)	0 (0.0)	0 (0.0)	0 (0.0)
Weight decreased	3 (1.2)	0 (0.0)	2 (0.8)	0 (0.0)
**Laboratory abnormalities**				
Aspartate aminotransferase increased	24 (9.5)	2 (0.8)	15 (5.9)	1 (0.4)
Alanine aminotransferase increased	15 (5.9)	2 (0.8)	7 (2.8)	1 (0.4)
Anaemia	13 (5.1)	8 (3.2)	8 (3.2)	5 (2.0)
Blood bilirubin increased	7 (2.8)	3 (1.2)	2 (0.8)	0 (0.0)
Neutropenia [a)]	7 (2.8)	5 (2.0)	7 (2.8)	5 (2.0)
Thrombocytopenia	4 (1.6)	2 (0.8)	4 (1.6)	2 (0.8)
Hypoalbuminaemia*	3 (1.2)	0 (0.0)	1 (0.4)	0 (0.0)

AE, adverse event; ADR, adverse drug reaction; CRC, colorectal cancer. Incidence rates are presented as number (%) of patients with AE and ADR. * Unexpected AE. [a)] Consolidated term comprising the following synonymous MedDRA preferred terms: neutropenia, neutrophil count decreased

Table S3. Incidence rates of AEs and ADRs for GIST patients

	GIST(N=14)			
	**AE**		**ADR**	
	Any grade No. (%)	≥ Grade 3 No. (%)	Any grade No. (%)	≥ Grade 3 No. (%)
**Clinical adverse event**				
Palmar-plantar erythrodysaesthesia syndrome	5 (35.7)	2 (14.3)	5 (35.7)	2 (14.3)
Asthenia	2 (14.3)	0 (0.0)	0 (0.0)	0 (0.0)
Constipation*	2 (14.3)	0 (0.0)	1 (7.1)	0 (0.0)
Decreased appetite	2 (14.3)	0 (0.0)	1 (7.1)	0 (0.0)
Diarrhoea	2 (14.3)	0 (0.0)	1 (7.1)	0 (0.0)
Myalgia	2 (14.3)	0 (0.0)	1 (7.1)	0 (0.0)
Abdominal pain	1 (7.1)	0 (0.0)	1 (7.1)	0 (0.0)
Acute kidney injury*	1 (7.1)	0 (0.0)	1 (7.1)	0 (0.0)
Anal abscess	1 (7.1)	0 (0.0)	1 (7.1)	0 (0.0)
Colitis*	1 (7.1)	0 (0.0)	1 (7.1)	0 (0.0)
Gastrointestinal haemorrhage	1 (7.1)	0 (0.0)	0 (0.00)	0 (0.0)
Insomnia*	1 (7.1)	0 (0.0)	0 (0.00)	0 (0.0)
Pneumonia	1 (7.1)	0 (0.0)	0 (0.00)	0 (0.0)
Pruritus*	1 (7.1)	0 (0.0)	1 (7.1)	0 (0.0)
Pyrexia	1 (7.1)	0 (0.0)	1 (7.1)	0 (0.0)
Rash	1 (7.1)	0 (0.0)	1 (7.1)	0 (0.0)

Upper respiratory tract infection	1 (7.1)	0 (0.0)	1 (7.1)	0 (0.0)
Urinary tract infection	1 (7.1)	0 (0.0)	0 (0.0)	0 (0.0)
Vomiting	1 (7.1)	0 (0.0)	0 (0.0)	0 (0.0)
**Laboratory abnormalities**				
Alanine aminotransferase increased	1 (7.1)	0 (0.0)	1 (7.1)	0 (0.0)
Aspartate aminotransferase increased	1 (7.1)	0 (0.0)	1 (7.1)	0 (0.0)

AE, adverse event; ADR, adverse drug reaction; GIST, gastrointestinal stromal tumors. Incidence rates are presented as number (%) of patients with AE and ADR. * Unexpected AE.

Table S4. Incidence rates of AEs and ADRs for HCC patients

	HCC(N=33)			
	AE		ADR	
	Any grade No. (%)	≥ Grade 3 No. (%)	Any grade No. (%)	≥ Grade 3 No. (%)
**Clinical adverse event**				
Palmar-plantar erythrodysaesthesia syndrome	5 (15.2)	0 (0.0)	5 (15.2)	0 (0.0)
Abdominal pain	4 (12.1)	1 (3.0)	3 (9.1)	0 (0.0)
Pyrexia	3 (9.1)	0 (0.0)	0 (0.0)	0 (0.0)
Abdominal distension*	2 (6.1)	2 (6.1)	0 (0.0)	0 (0.0)
Abdominal pain upper	2 (6.1)	0 (0.0)	2 (6.1)	0 (0.0)
Asthenia	2 (6.1)	1 (3.0)	1 (3.0)	0 (0.0)
Diarrhoea	2 (6.1)	0 (0.0)	1 (3.0)	0 (0.0)
Hyperkeratosis*	2 (6.1)	0 (0.0)	2 (6.1)	0 (0.0)
Melaena	2 (6.1)	1 (3.0)	1 (3.0)	0 (0.0)
Portal hypertension	2 (6.1)	0 (0.0)	0 (0.0)	0 (0.0)
Varices oesophageal*	2 (6.1)	2 (6.1)	0 (0.0)	0 (0.0)
Vomiting	2 (6.1)	0 (0.0)	2 (6.1)	0 (0.0)
Abdominal discomfort*	1 (3.0)	0 (0.0)	1 (3.0)	0 (0.0)
Anxiety*	1 (3.0)	0 (0.0)	0 (0.0)	0 (0.0)
Ascites*	1 (3.0)	0 (0.0)	0 (0.0)	0 (0.0)
Asterixis*	1 (3.0)	0 (0.0)	1 (3.0)	0 (0.0)

Chest pain	1 (3.0)	0 (0.0)	0 (0.0)	0 (0.0)
Cough*	1 (3.0)	0 (0.00)	0 (0.0)	0 (0.0)
Dizziness*	1 (3.0)	0 (0.00)	1 (3.0)	0 (0.0)
Fatigue	1 (3.0)	0 (0.00)	1 (3.0)	0 (0.0)
Haematuria	1 (3.0)	0 (0.00)	0 (0.0)	0 (0.0)
Headache	1 (3.0)	0 (0.00)	1 (3.0)	0 (0.0)
Hepatic encephalopathy*	1 (3.0)	0 (0.00)	0 (0.0)	0 (0.0)
Hepatorenal syndrome*	1 (3.0)	1 (3.03)	0 (0.0)	0 (0.0)
Nausea	1 (3.0)	0 (0.00)	1 (3.0)	0 (0.0)
Oedema*	1 (3.0)	0 (0.00)	0 (0.0)	0 (0.0)
Pneumonia	1 (3.0)	1 (3.03)	0 (0.0)	0 (0.0)
Rectal perforation	1 (3.0)	1 (3.03)	0 (0.0)	0 (0.0)
Renal injury*	1 (3.0)	0 (0.00)	0 (0.0)	0 (0.0)
**Laboratory abnormalities**				
Hypoalbuminaemia*	2 (6.1)	0 (0.0)	0 (0.0)	0 (0.0)

AE, adverse event; ADR, adverse drug reaction; HCC, hepatocellular carcinoma. Incidence rates are presented as number (%) of patients with AE and ADR. * Unexpected AE.

Table S5. Incidence rates of adverse events by dose modification

	CRC (N=254)	Total (N=301)
	Incidence rate<br>N (%)	Incidence rate<br>N (%)
No change	162 (63.8)	188 (62.5)
Dose reduction	61 (24.0)	71 (23.6)
Dose interruption	53 (20.9)	66 (21.9)
Permanent discontinuation	66 (26.0)	74 (24.6)

AE, adverse event; CRC, colorectal cancer.

Table S6. Incidence of adverse event by special interest populations

	**AE incidence,** N (%)			
	**CRC (N=254)**	**GIST (N=14)**	**HCC (N=33)**	**Total (N=301)**
**Elderly**				
≥65 years	59 (90.8)	2 (100.0)	11 (78.6)	72 (88.9)
<65 years	157 (83.1)	9 (75.0)	16 (84.2)	182 (82.7)
**p-value[a)]**	*0.1604*	*1.0000*	*1.0000*	*0.2146*
**Hepatic disorders**				
Yes	9 (81.8)	2 (100.0)	0 (0.0)	11 (84.6)
No	207 (85.2)	9 (75.0)	27 (81.8)	243 (84.4)
**p-value[a)]**	0.6719	*1.0000*	*NE*	*1.0000*
**Renal disorders**				
Yes	24 (85.7)	0 (0.0)	20 (90.9)	44 (86.3)
No	192 (85.0)	11 (84.6)	7 (63.6)	210 (84.0)
**p-value[a)]**	*1.0000*	*0.2143*	*0.1458*	*0.8333*
**Cardiovascular disorders**				
Yes	52 (81.3)	2 (66.7)	7 (70.0)	61 (79.2)
No	164 (86.3)	9 (81.8)	20 (87.0)	193 (86.2)
**p-value[a)]**	*0.3184*	*1.0000*	*0.3364*	*0.1500*
**Other concomitant disorders**				
Yes	129 (88.4)	4 (80.0)	7 (63.6)	140 (86.4)
No	87 (80.6)	7 (77.8)	20 (90.9)	114 (82.0)
**p-value[a)]**	*0.1089*	*1.0000*	*0.1458*	*0.3402*

AE, adverse event; CRC, colorectal cancer; GIST, gastrointestinal stromal tumors; HCC, hepatocellular carcinoma. [a)] P-value by Fisher’s exact test.

Table S7. Clinical parameters that affect CRC patients with and without dose modification or discontinuation* of regorafenib administration.

Variable	Reference	Parameter Estimate	Standard Error	OR	95% CI for OR	p-value [a)]
**Sex**	Female					
Male		-0.2172	0.2516	0.8048	(0.49, 1.32)	0.3880
**Age**	<70 years					
≥70 years		0.4659	0.3737	1.5934	(0.77, 3.31)	0.2125
**Number of metastatic sites**	1					
≥2		0.1516	0.2659	1.1637	(0.69, 1.96)	0.5686
**Metastatic site**	Other					
Liver		0.4431	0.2697	1.5575	(0.92, 2.64)	0.1004
**Prior therapy - radiotherapy**	No					
Yes		-0.2451	0.2797	0.7826	(0.45, 1.35)	0.3808
**Prior therapy - surgery**	No					
Yes		-0.06	0.3067	0.9417	(0.52, 1.72)	0.8448
**Prior treatment lines**	1-3					
≥4		-0.2138	0.4016	0.8075	(0.37, 1.77)	0.5945
**ECOG PS**	0-1					
2		0.9842	0.6017	2.6757	(0.82, 8.70)	0.1019
**Comorbidity**	No					
Yes		0.0717	0.2669	1.0744	(0.64, 1.81)	0.7881
**Response**	PD					
DCR		-0.2763	0.3173	0.7586	(0.41, 1.41)	0.3839
**Anti-EGFR biologics**	No					
Yes		-0.4884	0.2668	0.6136	(0.36, 1.04)	0.0672

CRC, colorectal cancer; OR, odds ratio; CI, confidence interval; ECOG PS, eastern cooperative oncology group performance status; PD, progressive disease; DCR, disease control rate; EGFR, estimated glomerular filtration rate. *Dose modification or discontinuation included dose change, dose interruption and permanent discontinuation [a)] P-value by Univariate logistic regression.
